# Effect of Doubled Sandblasting Process and Basic Simulated Body Fluid Treatment on Fabrication of Bioactive Stainless Steels

**DOI:** 10.3390/ma11081334

**Published:** 2018-08-01

**Authors:** Takeshi Yabutsuka, Ryoki Karashima, Shigeomi Takai, Takeshi Yao

**Affiliations:** 1 Department of Fundamental Energy Science, Graduate School of Energy Science, Kyoto University, Kyoto 606-8501, Japan; ryangki2337@gmail.com (R.K.); stakai@energy.kyoto-u.ac.jp (S.T.); 2 Institute of Advanced Energy, Kyoto University, Uji, Kyoto 611-0011, Japan; t_yao@hera.eonet.ne.jp

**Keywords:** stainless steels, hydroxyapatite-forming ability, ‘Basic SBF’ treatment, pores formation, sandblasting process, interlocking effect, surface roughness, surface area

## Abstract

In our recent study, we aimed to impart hydroxyapatite (HA)-forming to bioinert stainless steels (SUS316L). The surfaces of SUS316L specimen were treated by a sandblasting process using alumina grinding particles with 14.0 or 3.0 μm for average particle size, respectively. In addition, a doubled sandblasting process (DSP) using the 14.0 μm particles and subsequently 3.0 μm ones were also conducted. Compared with the case of the 14.0 μm particles, the 3.0 μm particles were available to increase the surface roughness and the surface area of the specimen. Moreover, these values were further increased in the case of the DSP. These specimens were soaked in simulated body fluid (SBF) at pH = 8.4, 25 °C and were directly heated in the solution by electromagnetic induction. By this treatment, formation of CaP was induced on each specimen. These materials performed high HA-forming ability in SBF. Average bonding strength of the HA film formed on them in SBF was increased depending on the increase of surface roughness and surface area. These results indicated that sandblasting condition was an important factor to improve interlocking effect related to the increase of the surface roughness and the surface area.

## 1. Introduction

Stainless steels are one of the most typical metallic biomaterials with high mechanical toughness and corrosion resistance. Among them, SUS316L, which is classified as an austenitic Fe-18Cr-12Ni-2.5Mo (0.03C) type stainless steel, is one of the extra-low carbon steels and has a strengthened intergranular corrosion resistance [1]. From these properties, SUS316L has been widely applied in the fields where corrosion resistance, mainly pitting one, are required such as a body environment. In fact, since the establishment of Charnley-type artificial hip joint, SUS316L has been widely used in clinical application. Recently, in addition, development of nickel free-type stainless steels for clinical use has been also progressed for the purpose of an improvement of low sensitizing properties in the body environment [2]. However, SUS316L does not have bone-bonding ability. If the bone-bonding ability is successfully imparted to SUS316L, bone restorative materials with high bone-bonding ability as well as excellent mechanical toughness and corrosion resistance can be developed.

In general, artificial substances grafted into the bone defect are spontaneously coated with non-calcified fibrous tissues and isolated from a surrounding living tissue. Such tissue formation is a normal immune reaction of the human body against exogenous substances. However, some-types of materials form a calcium-deficient hydroxyapatite (HA) film on the surface of them and can maneuver around the immune reaction. Consequently, the materials can adhere to bone through thus-formed HA film [3,4]. Such special materials property is often defined as ‘bioactivity’ in the research field of ceramic biomaterials. It is thought that the ‘bioactivity’ is one of the most important materials properties of bone-bonding materials.

Kokubo et al. reported that the HA formation reaction on the surface of the bioactive materials in the living body can be mimicked in simulated body fluid (SBF) whose ion concentrations and pH value are similar to those of human blood plasma [5,6,7,8]. By applying the Kokubo’s method, we can predict that the biomaterials performed such HA formation in SBF form HA film also in body environment and adhere to living bone through thus formed HA film.

As a typical method for providing bone-bonding ability to metallic materials, preparation of HA coatings by plasma spray method [9,10] have been widely applied in orthopedic and dental fields. In this method, however, it is reported that the formed HA film is heated over 10,000 °C in the manufacturing process and partially molten and decomposed. As a result, such HA films are not stable in living body for a long period [11]. Instead of the above method, SUS316L-HA composite prepared by hot-pressing technique [12], CO_2_ laser beam welding method [13], hopeite coating [14] and HA coating by thermally spray method [15] and so forth have been already reported as bioactivity treatment for SUS316L. However, bioactivity treatment to impart high HA-forming ability induced on the whole surface of the materials within 7 days in physiological SBF to SUS316L specimen has not been established yet.

In our previous study, we reported the production methodology of bioactive materials by an incorporation of amorphous calcium phosphate (ACP) to bioinert biomaterials [16]. By soaking the specimen with fine pores in SBF and raising its pH or temperature, nucleation of calcium phosphate (CaP) was accelerated and fine particles of ACP, named ‘apatite nuclei’ [17], were precipitated in the pores and on the surfaces. By this method, high bioactivity, that is, HA-forming ability, was successfully imparted to various kinds of bioinert biomaterials. In our previous studies, in addition, we invented the methodology for obtaining fine pores with complicated shape by the doubled sandblasting process (DSP) using different sizes of grinding ceramics particles on metals, ceramics and polymers [18]. When thus-treated specimens were treated with the above-mentioned bioactivity treatment, the HA film formed by soaking in SBF showed higher bonding strength in comparison with the case of the single sandblasting process (SSP) using one size of grinding ceramics particles by stronger interlocking effects [19]. However, details of a relationship between bonding strength of the formed HA film, combinations of grinding ceramics particles and roughness or area of the surfaces of specimen have not been investigated yet.

In our present study, we aimed to establish fabrication process of bioactive SUS316L by the incorporation of the CaP to SUS316L in SBF with basic condition. As a first step, we formed roughened surface on the SUS316L by the sandblasting method using various combination of the grinding ceramics particles. As a second step, we conducted the bioactivity treatment by the above-described methodology. HA-forming ability of the SUS316L was tested by soaking in physiological SBF and bonding strength of the HA film formed in the SBF was tested. By focusing the difference of surface roughness and surface area of the sandblasted SUS316L specimen, relationships between the sandblasting conditions and the bonding strength of HA film was evaluated.

## 2. Materials and Methods

### 2.1. Specimen

Commercially obtained SUS316L plates (Nisshin Steel Co., Ltd., Tokyo, Japan) with 15 × 10 × 2 mm^3^ in size were used as a specimen. This sample is defined as ‘S0’, hereinafter.

### 2.2. Sandblasting Process for Pores Formation

**Figure 1:**
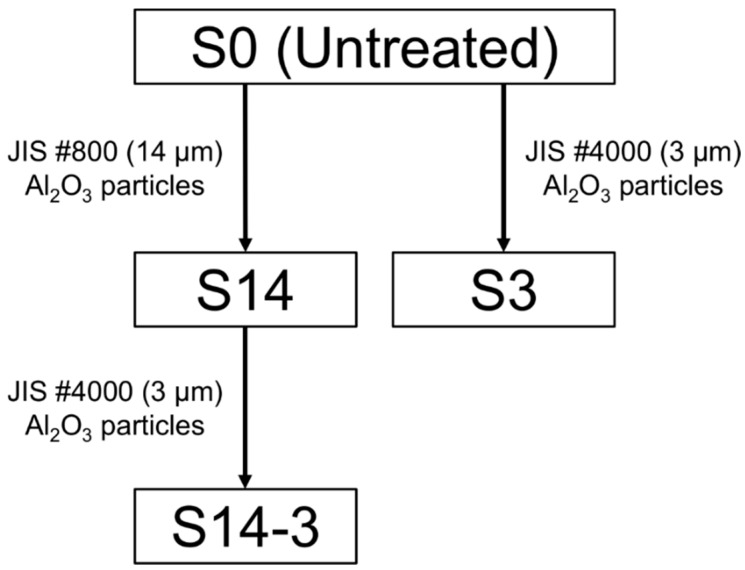
Flow chart of sandblasting processes and sample code for each condition.

The surfaces of S0 were treated with sandblasting process. Figure 1 shows a flow chart of sandblasting processes and sample code for each condition. The SUS316L were treated by the sandblasting process using alumina (Al_2_O_3_) particles with JIS #800 (14.0 ± 1.0 μm) or JIS #4000 (3.0 ± 0.4 μm) for particle size (WA, Fujimi Incorporated, Aichi, Japan) by using sandblasting machine (Fuji Manufacturing Co., Ltd., Tokyo, Japan). In the sandblasting, 850 kPa of discharge pressure was applied by an oil-free scroll compressor (SRL-3.7DMA5, Hitachi Industrial Equipment Systems Co., Ltd., Tokyo, Japan). The obtained samples are defined as ‘S14’ or ‘S3’, hereinafter. In addition, the SUS316L treated by the DSP, described in our previous reports [9,10], using the Al_2_O_3_ particles of the JIS #800 and subsequently ones of the JIS #4000 in size were also prepared. This sample is defined as ‘S14-3’, hereinafter. Thus-obtained S14, S3 and S14-3 were ultrasonically washed in acetone, ethyl alcohol and pure water and air-dried. Surface morphologies and element compositions of the surfaces were observed by field emission scanning electron microscopy (SEM; SU6600, Hitachi High-Technologies Corporation, Tokyo, Japan) and energy dispersive X-ray spectroscopy (EDS; Xflash^®^ 5010, Bruker AXS Inc., Fitchburg, WI, USA). Before the SEM/EDS observation, fine particles of gold (Au) were coated on the specimen by sputtering. The roughness, area and three-dimensional (3D) morphologies of the surface of the specimen were measured by laser microscopy (VK-9500, Keyence Corporation, Osaka, Japan). The surface roughness and surface area were calculated by the method certified as JIS B 0601 by using 3 samples for each sandblasting condition.

### 2.3. Preparation of SBF

SBF was prepared by the method reported by Kokubo et al. [6,7] and ISO 23317 [8] and buffered at pH = 7.6, 36.5 °C by disillusion of tris(hydroxymethyl)aminomethane (THAM; Hayashi Pure Chemical Ind., Ltd., Osaka, Japan).

### 2.4. ‘Basic SBF’ Treatment

The pH of SBF were raised to pH = 8.4 by dissolution of THAM at 25 °C. This solution is defined as ‘Basic SBF’, hereinafter. A gum tube was infused with the ‘Basic SBF’ and S14, S3 and S14-3 were soaked in the ‘Basic SBF.’ In order to penetrate ‘Basic SBF’ in the pores of the specimen, a hydrostatic press was applied at 392 MPa for 1 h by using a cold isostatic press machine (CIP-SI, Kobe Steel, Ltd., Kobe, Japan). Then, the specimen located in the ‘Basic SBF’ were heated by electromagnetic induction at 2500 W for 180 min by using an induction heater (KZ-KM22B, Panasonic Corporation, Osaka, Japan). Surface morphologies and element compositions of the specimen were observed by SEM/EDS. Crystal phase of the surfaces of the specimen was examined by thin film X-ray diffractometry (XRD, Rint 2500, Rigaku Corporation, Tokyo, Japan) using CuKα radiation at 50 kV, 0.3 A.

### 2.5. Test of HA-Forming Ability

HA-forming ability of each specimen was tested by soaking in physiological SBF (pH = 7.4 at 36.5 °C). After the soaking in SBF for 1 day, 3 days and 7 days, the specimen were rinsed with pure water and air-dried. Surface morphologies and element compositions of the specimen were observed by SEM/EDS. Crystal phase of the surfaces of the specimen were examined by XRD.

### 2.6. Test of Bonding Strength of HA Film Formed in SBF

The ‘Basic SBF’ treatment mentioned in 2.4 was conducted for S0, S14, S3 and S14-3. HA coatings were conducted for each condition of specimen by soaking in SBF for 14 days. Bonding strength between the specimen and the formed HA film was examined by a modified ASTM C633 [20,21,22,23]. SUS jigs (10 × 10 mm^2^) were adhered to surfaces of the specimen using Araldite^®^ glue (Nichiban Co., Ltd., Tokyo, Japan) and tensile load was applied at 6 cm∙h^−1^ of cross-head speed until fracture were caused between HA films and the specimen using universal testing machine (Model AGS-H Autograph, Shimadzu Corporation, Kyoto, Japan).

## 3. Results and Discussion

### 3.1. Effect of Sandblasting Process

**Figure 2:**
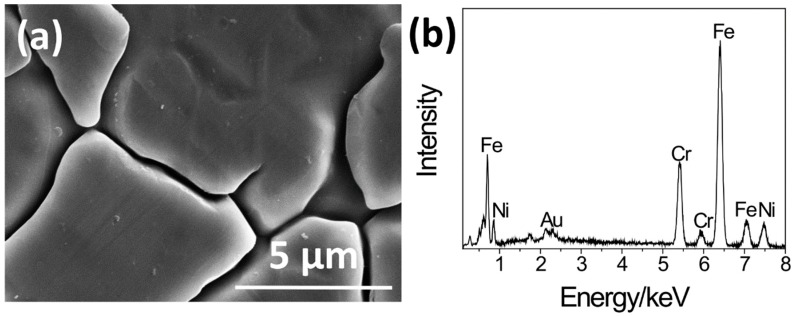
(**a**) SEM picture and (**b**) EDS spectrum of the surface of S0.

Figure 2 shows the SEM picture and the EDS spectrum of the surface of the S0. In the SEM picture, grain boundaries obtained by a manufacturing process were observed on the whole surface. In the EDS spectrum, peaks of iron (Fe), chromium (Cr) and nickel (Ni), constituents of SUS316L, were observed.

**Figure 3:**
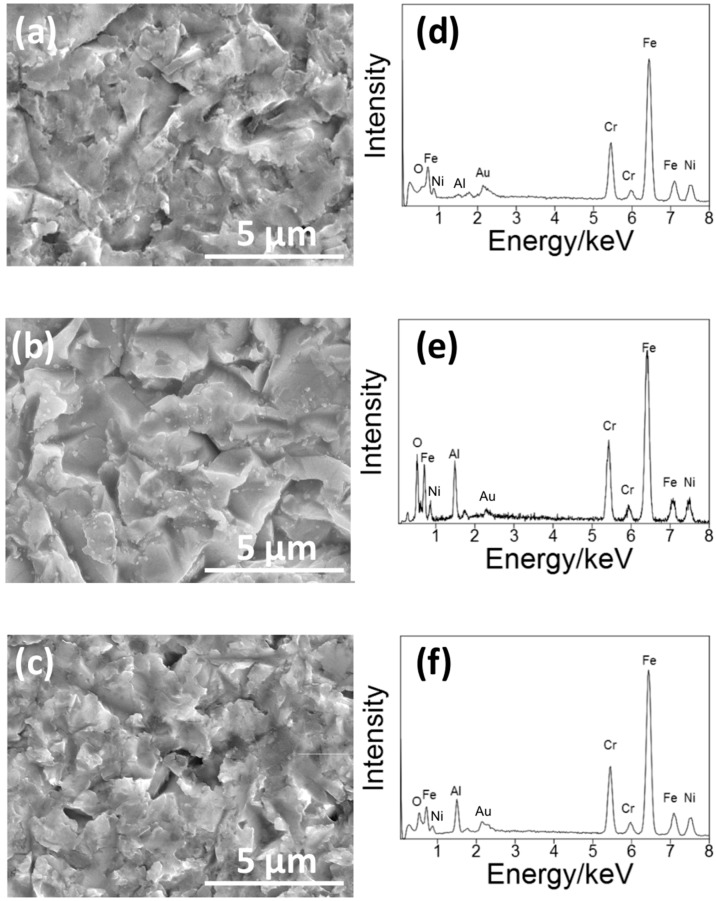
(**a**–**c**) SEM pictures and (**b**–**d**) EDS spectra of the surface of (**a**,**d**) S14, (**b**,**e**) S3 and (**c**,**f**) S14-3.

Figure 3 shows the SEM pictures and the EDS spectra of the surface of the S14, S3 and S14-3. After each condition of sandblasting processes, surface morphologies showed characteristic changes in the case of the sandblasting process. Pores in micron-scale were observed on the whole surface on each specimen. In the EDS spectra, peaks of aluminum (Al) were observed on each specimen. The Al peaks were attributed to Al_2_O_3_ grinding ceramics particles remained near the surface. This result means that the Al_2_O_3_ particles grinded the surfaces of each SUS316L specimen in the sandblasting processes. However, the difference of surface morphologies between each sandblasting condition was not clearly observed by the SEM observation. To clarify the difference of the surface morphology between each sandblasting condition, the authors also conducted the laser microscope observation mentioned in the next paragraph.

**Figure 4:**
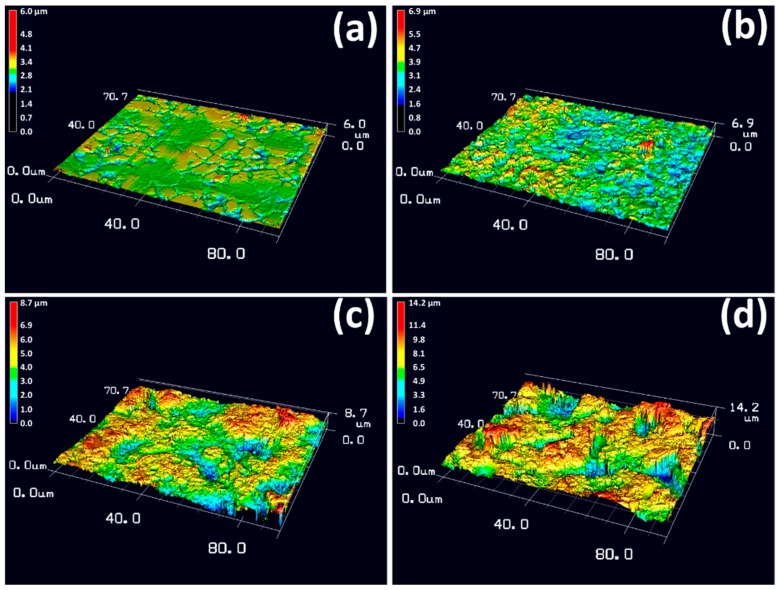
Three dimensional images of the surface of (**a**) S0, (**b**) S14, (**c**) S3 and (**d**) S14-3.

Figure 4 shows the 3D surface morphologies of S0, S14, S3 and S14-3 observed by the laser microscope. In Figure 4a, it was observed that S0 possessed grain boundaries obtained by manufacturing processes on the whole surface. In Figure 4b–d, it was observed that surface of the specimen was roughened by the sandblasting process in comparison with S0. Although the difference of the surface morphology was not clarified in SEM observation, the difference was clarified by the 3D observation. The surface of S14 was roughened compared with S0 but the degree of roughness was the lowest among the three-types of sandblasting condition. In contrast, the degree of roughness of S14-3 was the highest among each condition.

**Figure 5:**
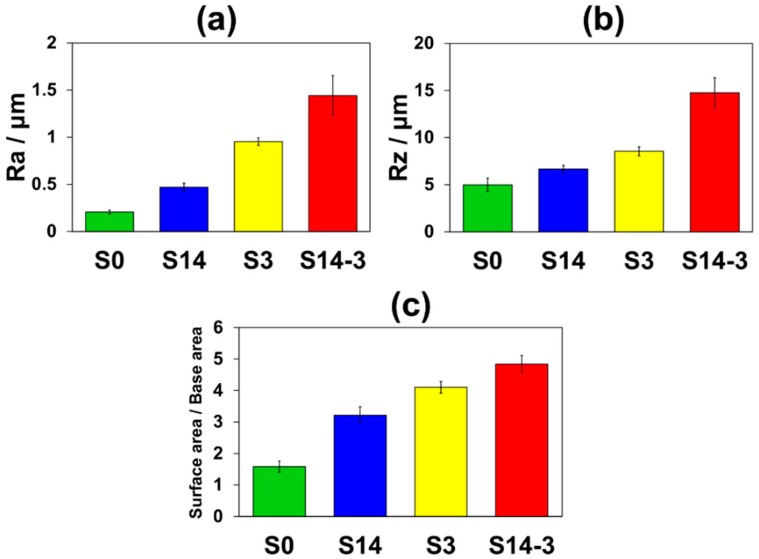
(**a**) R_a_ and (**b**) R_z_ in surface roughness and (**c**) values obtained by dividing surface areas by base areas for S0, S14, S3 and S14-3. Error bars show the standard deviations.

Figure 5 shows the surface roughness (R_a_ and R_z_) and the values obtained by dividing surface areas by base areas of S0, S14, S3 and S14-3. In Figure 5, average values were calculated by using 3 samples and error bars show their standard deviations. It was observed that S0 possessed smallest surface roughness and surface area among the four-types of specimen. In comparison S14 with S3, S3 showed larger surface roughness and surface area than those of S14. This result indicates that smaller grinding ceramics particles were available for attainment of larger roughness and area of the surface. In addition, S14-3 showed the largest roughness and area of the surface among the four types of specimen. This result indicates that the DSP with different size of the grinding ceramics particles was effectively contributed to achieve larger surface roughness and surface area than the SSP.

### 3.2. Effect of ‘Basic SBF’ Treatment

**Figure 6:**
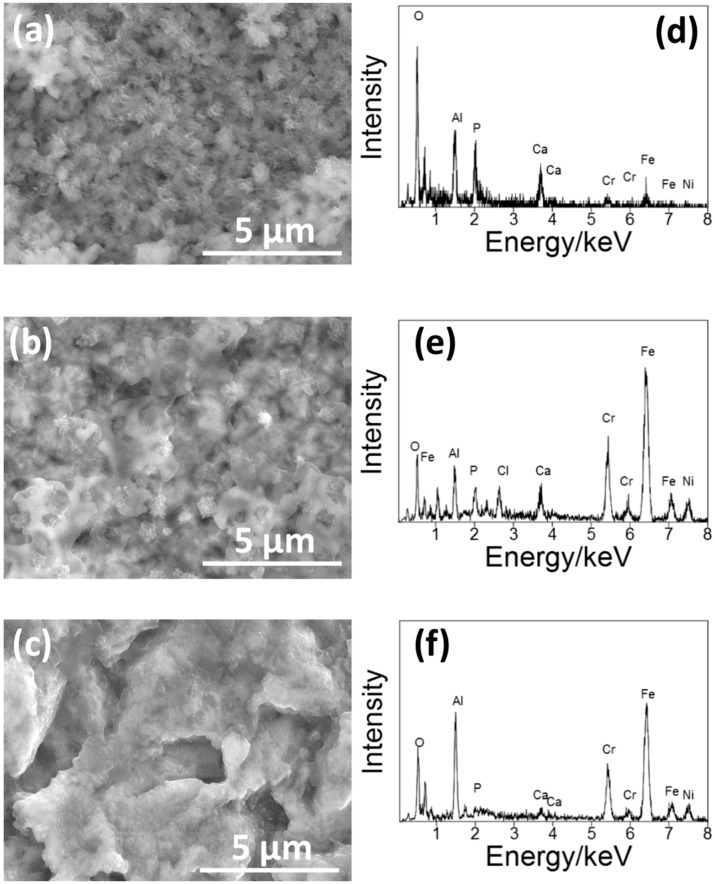
(**a**–**c**) SEM picture and (**d**–**f**) EDS spectra of the surface of (**a**,**d**) S14, (**b**,**e**) S3 and (**c**,**f**) S14-3 after the ‘Basic SBF’ treatment.

Figure 6 shows the SEM picture of the surface of S14, S3 and S14-3 after ‘Basic SBF treatment’. After the ‘Basic SBF’ treatment, film-like precipitates were deposited on the whole surface of each specimen. In the EDS spectra, peaks of calcium (Ca) and phosphorus (P), constituents of CaP, were observed on each specimen. From this result, it is considered that some type of CaP film was formed on the whole surface of each specimen by ‘Basic SBF’ treatment.

**Figure 7:**
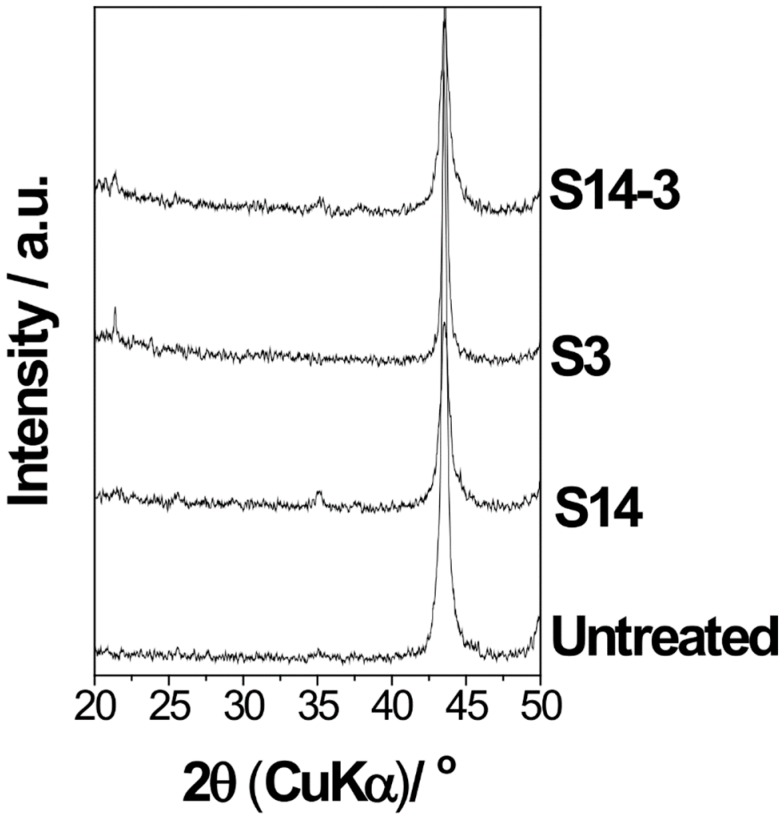
XRD patterns of the surface of S14, S3 and S14-3 after the ‘Basic SBF’ treatment and the untreated SUS316L plate.

Figure 7 shows the XRD patterns of the surface of S14, S3 and S14-3 after ‘Basic SBF treatment’. In Figure 7, it is considered that several numbers of peaks observed on the specimen after the sandblasting process was attributed to impurities such as remained grinding ceramics particles. On the other hand, diffraction peaks of HA, located around 2θ = 26° and 31°, were not observed. Taking into consideration the results of the SEM, EDS and XRD, it is suggested that the CaP formed by ‘Basic SBF’ treatment possessed low crystallinity and consisted largely of amorphous phase.

These results suggested two points described in below. First point is about the effect of high pH value on CaP formation. The increasing pH value of SBF promoted CaP-forming reaction. The formation of HA in an aqueous solution can be described by the following chemical equilibrium.

10Ca^2+^ + 6PO_4_^3−^ + 2OH^−^ = Ca_10_(PO_4_)_6_(OH)_2_ (1)

Taking into consideration the above chemical equilibrium, an ionic activity product (IP) of HA is given by the following formula where ‘γ’ is the activity coefficient and ‘[ ]’ is the concentration of each ion.

IP = (γCa^2+^)^10^(γPO_4_^3−^)6(γOH^−^)^2^ × [Ca^2+^]^10^[PO_4_^3−^]^6^[OH^−^]^2^ (2)

It is reported that the conventional SBF with physiological condition, that is, pH 7.40 at 36.5 °C, is supersaturated against HA. Generally, however, HA formation in SBF is induced only on the surface of specified materials, that is, bioactive materials, because of high energetic obstacles toward the HA formation. By raising pH of the aqueous solution, on the other hand, the IP is increased because of the increase of OH^−^ concentration. Hence, it is suggested that high pH environment in ‘Basic SBF’ treatment removed the energetic obstacles. Moreover, it is considered that the SBF located adjacent to the surface of the specimen was effectively heated by the induction heating of the SUS316L and formation of CaP was accelerated near the surface of the specimen.

Second point is about the effect of additional minute minerals such as magnesium and carbonic acid in SBF to the crystallinity of the formed CaP. In the ‘Basic SBF’ treatment in this study, a progress of crystallization was insufficient and the formed CaP film was consisted largely of amorphous phase rather than crystalline HA as shown in Figure 7. It is considered that this is because the ion composition of SBF. Okazaki et al. reported that the existence of magnesium and carbonic acid inhibit crystallization of HA [24,25]. Hence, it is speculated that this was because that SBF contains additional several kinds of minute minerals such as magnesium and carbonic acid besides calcium and phosphorous and these minute minerals considerably inhibited the crystallization of CaP.

### 3.3. HA-Forming Ability

**Figure 8:**
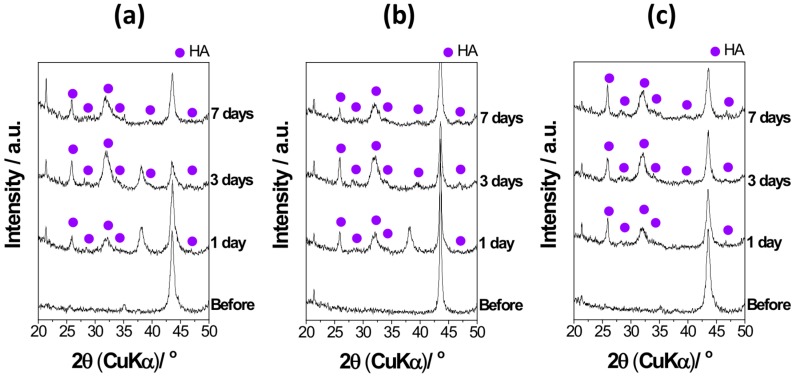
XRD patterns of (**a**) S14, (**b**) S3 and (**c**) S14-3 after ‘Basic SBF’ treatment (Before) and subsequently soaking in SBF for 1 day, 3 days and 7 days.

Figure 8 shows the XRD patterns of the surface of S14, S3 and S14-3 after ‘Basic SBF’ treatment and subsequently soaking in SBF for 1 day, 3 days and 7 days. After the soaking in SBF for 1 day, diffraction peaks of HA with broad shapes were appeared around 2θ = 26°, 28°, 34° and 47°. After the soaking in SBF for 3 days, the number of diffraction peaks of HA increased in comparison with the case of 1 day. From the XRD results, it is considered that the CaP with low crystallinity was grew into crystalline HA within 1 day in SBF and high HA-forming ability was attained.

**Figure 9:**
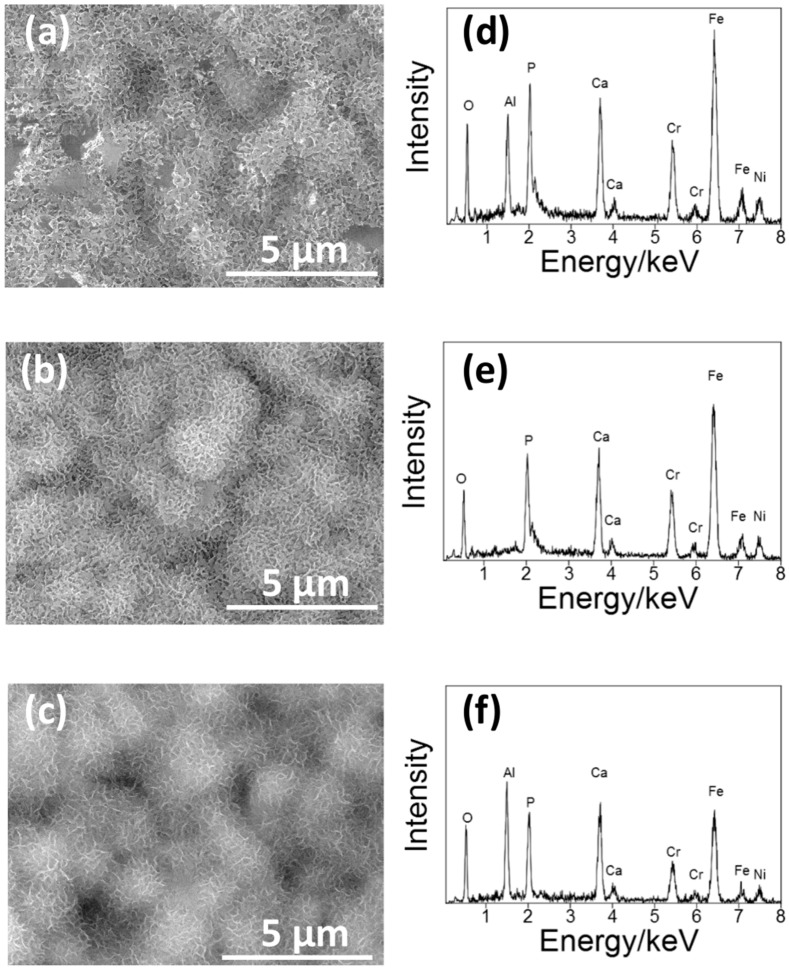
(**a**–**c**) SEM pictures and (**d**–**f**) EDS spectra of the surface of (**a**,**d**) S14, (**b**,**e**) S3 and (**c**,**f**) S14-3 after ‘Basic SBF’ treatment and subsequently soaking in SBF for 1 day.

Figure 9 shows the SEM pictures and the EDS spectra of the surface of S14, S3 and S14-3 after ‘Basic SBF’ treatment and subsequently soaking in SBF for 1 day. In the SEM observation, flake-like crystallites characteristic to HA formed in SBF, so-called ‘bone-like HA,’ covered the whole surface of each specimen. Considerable difference of HA formation was not observed among each sandblasting condition. In the EDS analysis, peaks of Ca and P, which are constituents of HA, were clearly observed on each specimen. Taking into consideration the results of the XRD, SEM and EDS, it is revealed that the CaP film with low crystallinity formed in ‘Basic SBF’ treatment was crystallized in the conventional SBF and the induced HA grew on the whole surface of each SUS316L specimen within 1 day. This means that high HA-forming ability was performed on the bioinert SUS316L by conducting ‘Basic SBF’ treatment. In the case of bioactivity treatment such as SUS316L-HA composite prepared by hot-pressing technique [12], CO_2_ laser beam welding method [13], hopeite coating [14] and HA coating by thermally spray method [15] and so forth, it was reported that their HA-forming ability was induced within approximately 2–4 weeks. This result suggests that the ‘Basic SBF’ treatment in the present study is effective for acceleration of the HA formation.

### 3.4. Bonding Strength of HA Film

**Figure 10:**
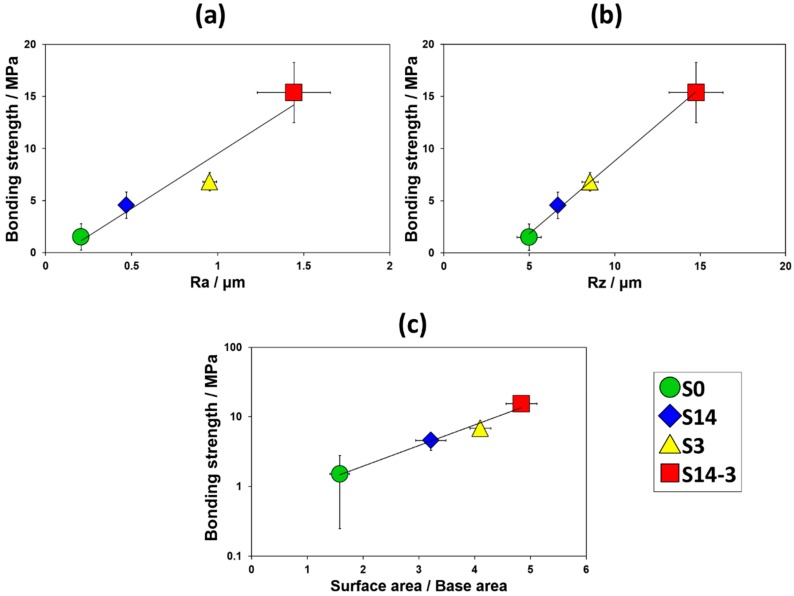
Changes in the bonding strength toward (**a**) R_a_ and (**b**) R_z_ in the surface roughness and (**c**) the value obtained by dividing the surface areas by the base areas.

Figure 10 shows the changes in the bonding strength against the surface roughness (R_a_ and R_z_) and the value obtained by dividing the surface areas by the base areas. The average bonding strengths of HA films and the standard deviations were 1.51 (1.26) MPa for S0 (10 samples), 4.57 (1.27) MPa for S14 (9 samples), 6.81 (0.88) MPa for S3 (7 samples) and 15.37 (2.90) MPa for S14-3 (8 samples), respectively. Toward the surface roughness, the average bonding strength of HA film monotonically and linearly increased. Toward the surface area, in addition, the average bonding strength of HA film monotonically and exponentially increased. As the surface roughness or the surface area was increased, interlocking effect was effectively achieved and bonding strength of HA film was enhanced. In Figure 4 mentioned in the above paragraph, the authors described that the particle size and the combination of the grinding ceramics particles in the sandblasting process was tightly related to the surface roughness and the surface area of the surface of the specimen. From the viewpoint of such relationship between the combination of grinding ceramics particles in the sandblasting process and the surface roughness or the surface area, these results revealed that the sandblasting condition which achieved high these values is an important factor for enhancing the interlocking effect. As one of the solutions for this problem, the DSP has a possibility to be a candidate for achievement of high bonding strength of the HA film formed in body environment.

Qu et al. reported that they formed HA films on sandblasted, gritted and acid-etched titanium metal by aqueous solution method using SBF with modified ion composition [26]. They reported that bonding strength of the formed HA film was approximately 8–18 MPa. In comparison with the Qu’s result, the bonding strength of HA film formed on the surface of at least S14-3, fabricated by DSP in our study, was almost equivalent. Hence, it is speculated that bonding strength of HA films formed on metallic materials in aqueous solution show almost such values. In contrast, Liu et al. reported that bonding strength of HA film formed on the surface of SUS316L by sol-gel method was approximately 40–50 MPa [27]. However, such fabrication method is required annealing process. Our result suggests that even HA film induced in biomimetic environment, that is, aqueous solution under normal temperature and pressure, have a possibility to improve bonding strength to bioinert SUS316L by adjusting sandblasting condition for increase of surface roughness and surface area.

We acknowledge that there are several limitations in this study. Firstly, the CaP formed in the ‘Basic SBF’ treatment possessed variety in shape among each sandblasting condition. However, the specimen showed HA-forming ability within 1 day irrespective of the sandblasting condition, shapes and crystallinities of the formed HA was similar and the bonding strength was tightly related to the surface roughness and surface area. From this reason, the authors think that this fabrication method is reasonable for providing HA-forming ability to SUS316L. The authors think that the control of the shapes and the crystallinities of the CaP formed in the ‘Basic SBF’ treatment is our next-step theme. Secondly, the authors have not conducted cross-sectional study mainly about the specimen after HA formation in SBF yet. It is thought that the interlocking effect can be effectively performed when the HA is formed inside the pores deeply. In order to clarify this point, cross-sectional observations of the specimen are needed. If the formed HA is thickly meshed inside the pores, both strong interlocking effects, which contributes to fixation in the bone defects and inhibition of bacterial growth at the interface on the specimen, which contributes to reduction of infection risks living body, is expected. Thirdly, the authors applied only two sizes of grinding particles in this study. In order to discuss the relationship between surface roughness and bonding strength of HA film more statistically, we think that it is needed to increase the kinds of grinding particles with different particle size. In addition, operation times and discharge pressure of sandblasting process will also affect to the surface roughness and the bonding strength. For evaluation of bioactivity, finally, test in not only acellular biomimetic environment such as SBF but also ex vivo or tissue environment is needed. These points will be clarified in our future studies.

## 4. Conclusions

We prepared bioactive SUS316L by forming roughened surface on the SUS316L by the sandblasting method and subsequently conducted ‘Basic SBF’ treatment. By the soaking of the bioactive SUS316L in SBF, the whole surface of the bioactive SUS316L was covered with HA film within 1 day. The formed HA film showed high bonding strength to the SUS316L specimen by the interlocking effect. This value was tightly related to the surface roughness and the surface area of the surface of the specimen. This means that the sandblasting condition is an important factor for enhancement of the bonding strength of the formed HA film. This material is promising as one of the novel implant materials with high HA-forming ability, that is, bioactivity, as well as high mechanical toughness for clinical applications.
